# Right ventricular failure following left ventricular assist device implantation is associated with a preoperative pro-inflammatory response

**DOI:** 10.1186/s13019-019-0895-x

**Published:** 2019-04-25

**Authors:** Paul C. Tang, Jonathan W. Haft, Matthew A. Romano, Abbas Bitar, Reema Hasan, Maryse Palardy, Keith D. Aaronson, Francis D. Pagani

**Affiliations:** 1 Department of Cardiac Surgery, University of Michigan Frankel Cardiovascular Center, 5158 Cardiovascular Center, SPC 5864, 1500 E. Medical Center Drive, Ann Arbor, MI 48109-5864 USA; 2 Division of Cardiovascular Medicine, University of Michigan Frankel Cardiovascular Center, Ann Arbor, MI USA

**Keywords:** Inflammation, Left ventricular assist device, Right ventricular failure, Heart failure

## Abstract

**Background:**

Systemic inflammation during implant of a durable left ventricular assist device (LVAD) may contribute to adverse outcomes. We investigated the association of the preoperative inflammatory markers with subsequent right ventricular failure (RVF).

**Materials and methods:**

Prospective data was collected on 489 patients from 2003 through 2017 who underwent implantation of a durable LVAD. Uni- and multivariable correlation with leukocytosis was determined using linear and binary logistic regression. The population was also separated into low (< 10.5 K/ul, *n* = 362) and high (> 10.5 K/ul, *n* = 127) white blood cell count (WBC) groups. Mantel-Cox statistics was used to analyze survival data.

**Results:**

Postop RVF was associated with a higher preop WBC (11.3 + 5.7 vs 8.7 + 3.1) and C-reactive protein (CRP, 5.6 + 4.4 vs 3.3 + 4.7) levels. Multivariable analysis identified an independent association between increased WBC preoperatively with increased lactate dehydrogenase (LDH, *P* < 0.001), heart rate (*P* < 0.001), CRP (*P* = 0.006), creatinine (*P* = 0.048), and INR (*P* = 0.049). The high WBC group was more likely to be on preoperative temporary circulatory support (17.3% vs 6.4%, P < 0.001) with a trend towards greater use of an intra-aortic balloon pump (55.9% vs 47.2%, *P* = 0.093). The high WBC group had poorer mid-term survival (*P* = 0.042).

**Conclusions:**

Postop RVF is associated with a preoperative pro-inflammatory environment. This may be secondary to the increased systemic stress of decompensated heart failure. Systemic inflammation in the decompensated heart failure may contribute to RVF after LVAD implant.

## Introduction

Inflammatory mediators are known to contribute to the development and progression of heart failure [1, 2] In the setting of durable left ventricular assist device (LVAD) implantation, postoperative right ventricular failure (RVF) complicates 9–44% of implants. This is associated with a greater incidence of morbidity, decreased survival, greater demands on resources and worse long term outcomes [3, 4]. While the majority of studies have focused on the hemodynamic contributions to the right ventricular dysfunction (RVD) [5], the etiology of RVF is likely a complex interaction of mechanical burden with underlying ventricular biology.

There is gathering evidence to implicate the immune response in heart failure pathophysiology [6]. Decreased chemokine receptor expression has been associated with a greater incidence of right heart failure following durable mechanical circulatory support (MCS), and greater mortality at 1 year [7]. Pre-LVAD inflammatory status also has significant prognostic implications. Higher IL-6 levels post-LVAD implantation is associated with longer ICU stay, hospitalization and higher Sequential Organ Failure Assessment (SOFA) score after 1 week correlating with greater multi-organ dysfunction [8]. Postoperatively, elevated IL-6 levels during the early post-LVAD implant period was associated with death from multi-organ dysfunction, and monocyte activation was proposed as a critical mechanism [9].

While these cytokine and chemokine markers of inflammation are important for prognosis, they have limited availability in a clinical setting. There is also scarce long term follow up data associated with these inflammatory markers. Therefore, this study examines the white blood count (WBC) and C-reactive protein (CRP), two commonly measured lab values, to determine their prognostic value for patients undergoing durable LVAD implantation.

## Methods

### Patients

This study was approved by the University of Michigan Institutional Review Board (IRB#HUM00135533). A waiver of informed consent was approved by the University of Michigan IRB. We conducted a retrospective review of prospectively collected data from 489 total patients from the University of Michigan Mechanical Circulatory Support Registry (IRB#HUM00020274) who underwent continuous flow LVAD (cfLVAD) implantation at the from January 1, 2003 to June 1, 2017. We separated patients into a preoperative groups of low WBC (< 10.5 K/uL, *n* = 362) and high WBC (> 10.5 K/uL, *n* = 127). We excluded patients who underwent pulsatile durable LVADs in our study since these have been largely replaced by pulsatile flow devices and carry a very different postoperative outcomes and complication profile. We included only patients aged 18 years or older and also included patients who needed postoperative biventricular support.

### Follow up

Survival and heart transplant data was available for all 489 patients in this study who underwent cfLVAD implantation. These were obtained through detailed prospective clinical follow-up. Censoring was performed at the time of heart transplantation or device explant (without replacement) for myocardial recovery or device complication or device inactivation (device not removed). Longest follow-up was 12.8 years with a total follow up of 996.8 patient years. Median follow up was 1.32 (IQR = 2.25) years with a mean follow up of 2.0 + 2.1 years.

Right ventricular failure defined as a central venous pressure > 18 mmHg with a cardiac index < 2.0 l/min/m2 in the absence of tamponade, ventricular arrhythmias or pneumothorax requiring RVAD or inhaled pulmonary vasodilator (e.g. nitric oxide) or inotropic therapy for > 1 week at any time following LVAD implantation [10]. Only postoperative RVF occurring during the index hospitalization following LVAD implantation was considered. Device infection is per criteria established by the Interagency Registry for Mechanically Assisted Circulatory Support (INTERMACS) [11]. Briefly, cutaneous site infections are defined as pain, erythema or purulent drainage at the driveline site with positive skin cultures. LVAD infection are identified with positive blood cultures combined with fever and leukocytosis not explained by other potential causes.

### Statistical methods

The Pearson chi square test or Fisher’s exact test was used to analyze categorical variables. Student’s t-test was used to compare continuous variables. Uni- and multivariable correlation with leukocytosis was determined using linear and binary logistic regression. Kaplan-Meier Survival analysis with Mantel-Cox statistics was used to analyze survival data. Statistical analysis was performed using the Statistical Package for the Social Sciences software (SPSS Inc., Chicago, IL).

## Results

### Preoperative laboratory parameters and patient demographics

Patients with preoperative WBC > 10.5 K/μL had a mean WBC of 13.20 + 3.56 and those with WBC < 10.5 K/μL had a mean WBC of 7.38 + 1.65 (Table 1, *P* < 0.001). The high WBC group also had a higher level of another inflammatory marker CRP (*P* < 0.001). The lower serum Na^+^ in this group (*P* = 0.004) was also consistent with a worse degree of heart failure. Greater hepatic impairment was also observed in the in the high WBC group along with a higher total bilirubin (*P* = 0.005), PT (*P* = 0.002) and INR (*P* = 0.013). No difference in preoperative serum creatinine (*P* = 0.273) or mixed venous oxygen saturation (*P* = 0.953) was seen (Table 1). Interestingly, the high WBC group was younger (51.21 + 13.01 vs 56.53 + 12.96, *P* < 0.001) and had a higher BMI (*P* = 0.005). No differences were seen in other demographic variables or comorbidities (*P* > 0.05) although there was a trend toward greater use of preoperative CRT-D in the high WBC group (*P* = 0.063) (Table 1).

**Table 1:** Patient Demographics and Preoperative Laboratory Results

	WBC < 10.5 K/μL (*n* = 362)	WBC > 10.5 K/μL (*n* = 127)	*P* Value
Age (years)	56.53 + 12.96	51.21 + 13.01	<0.001
Male	284 (78.5%)	99 (78.0%)	0.906
Height (cm)	174.79 + 9.76	173.50 + 10.13	0.205
Weight (kg)	84.72 + 20.87	87.91 + 23.66	0.154
BMI	27.61 + 5.99	29.62 + 9.04	0.005
Hypertension	179 (49.4%)	54 (42.5%)	0.179
Diabetes	125 (34.5%)	41 (32.3%)	0.645
Stroke or TIA	44 (12.2%)	16 (12.6%)	0.896
Carotid Disease	27 (7.5%)	8 (6.3%)	0.663
Hyperlipidemia	222 (61.3%)	71 (55.9%)	0.284
Atrial Fibrillation	81 (22.4%)	28 (22.0%)	0.939
Dialysis	0 (0.0%)	1 (0.8%)	0.091
ICD	305 (84.3%)	100 (78.7%)	0.156
CRT-D	180 (52.5%)	53 (42.7%)	0.063
WBC (K/uL)	7.38 + 1.65	13.20 + 3.56	<0.001
CRP (mg/dL)	2.80 + 4.05	5.29 + 6.04	<0.001
Hb (g/dL)	11.79 + 8.14	11.22 + 2.18	0.433
Na (mmol/L)	134.30 + 4.39	132.95 + 4.92	0.004
Cl (mmol/L)	99.28 + 5.69	96.24 + 6.29	<0.001
HCO3 (mmol/L)	27.72 + 3.76	28.76 + 4.39	0.010
BUN (mg/dL)	29.73 + 13.90	34.58 + 17.17	0.002
Cr (mg/dL)	1.31 + 0.51	1.37 + 0.50	0.273
BNP (pg/mL)	911.52 + 996.64	983.53 + 1034.26	0.493
Alk Phos (IU/L)	100.40 + 43.96	102.63 + 59.18	0.655
Total Bili (mg/dL)	1.11 + 0.67	1.43 + 1.21	0.005
PT (seconds)	11.61 + 1.11	12.05 + 1.88	0.002
INR	1.11 + 0.13	1.16 + 0.20	0.013
Uric Acid (mg/dL)	8.36 + 2.90	9.05 + 3.17	0.035
SVO2 (%)	55.65 + 8.55	55.59 + 9.52	0.953

Continuous variables are mean + standard deviation

### Cardiac and hemodynamic characteristics

The high WBC group (Table 2) had a higher heart rate (94.91 + 19.11 vs 86.13 + 16.72, *P* < 0.001 bpm), right atrial pressure (9.84 + 5.29 vs 8.63 + 4.88 mmHg, *P* = 0.019) and a lower right ventricular stroke work index (551.5 + 226.7 vs 615.5 + 269.1 g/m/m^2^/beat, *P* = 0.011). The high WBC group had a higher acuity INTERMACS profile (Table 2). There was no difference in left ventricular ejection fraction, right ventricular function or valvular characteristics between the 2 groups (*P* > 0.05). The high WBC group (Table 3) was more likely to be bridged to transplant (70.1%) while the low WBC group was more likely to be destination therapy (41.7%, *P* = 0.019). Patients in the high WBC group were more likely to be on preoperative temporary circulatory support (TCS, 17.3% vs 6.4%, *P* < 0.001) and be on it for a longer period of time (6 vs 4 days, *P* = 0.022). Similarly, the high WBC group had a trend for more IABP (55.9% vs 47.2%, *P* = 0.093), and also for a longer duration (median of 2 vs 1 days, *P* = 0.012). Multivariable analysis identified an independent association between increased WBC preoperatively with increased LDH (*R* = 0.389, *P* < 0.001), heart rate (*R* = 0.243, *P* < 0.001), CRP (R = 0.345, *P* = 0.006), creatinine (*R* = 0.119, *P* = 0.048), and INR (*R* = 0.164, *P* = 0.049).

**Table 2:** Preoperative Hemodynamics and INTERMACS Class

	WBC < 10.5 K/μL (*n* = 362)	WBC > 10.5 K/μL (*n* = 127)	*P* Value
HR (bpm)	86.13 + 16.72	94.91 + 19.11	<0.001
Mean Arterial BP (mmHg)	74.96 + 8.84	76.38 + 9.97	0.133
CO (L/min)	4.49 + 1.18	4.69 + 1.49	0.121
CI (L/min/m^2^)	2.25 + 0.55	2.32 + 0.67	0.291
PCWP (mmHg)	19.74 + 6.72	20.85 + 7.35	0.122
PAP (mmmHg))	31.50 + 8.14	32.58 + 8.39	0.202
RAP (mmHg)	8.63 + 4.88	9.84 + 5.29	0.019
PVR (Wood Units)	2.78 + 1.38	2.84 + 1.69	0.704
SVR (dynes/sec/cm^5^)	1265.88 + 392.30	1253.54 + 478.32	0.776
TPG (mmHg)	11.76 + 4.83	11.70 + 4.81	0.899
RVSWI (gm/m/m^2^/beat)	615.46 + 269.14	551.54 + 226.69	0.011
CVP/PCWP Ratio	0.44 + 0.23	0.48 + 0.25	0.124
INTERMACS			
1	37 (10.2%)	35 (27.6%)	<0.001
2	90 (24.9%)	39 (30.7%)	0.198
3	188 (51.9%)	40 (31.5%)	<0.001
4	47 (13.0%)	13 (10.2%)	0.417

Continuous variables are mean + standard deviation

**Table 3:** Preoperative Echocardiographic Findings, and Mechanical Support

	WBC < 10.5 K/μL (n = 362)	WBC > 10.5 K/μL (n = 127)	*P* Value
LVEF (%)	15.33 + 5.78	15.40 + 5.60	0.900
Moderate-Severe RVD	182 (50.3%)	73 (57.3%)	0.162
Severe AI	14 (3.9%)	2 (1.6%)	0.211
Severe MR	123 (34.0%)	40 (31.5%)	0.610
Severe TR	53 (14.6%)	14 (11.0%)	0.308
Bridge to Txp	211 (58.3%)	89 (70.1%)	0.019
Destination	151 (41.7%)	38 (29.9%)	0.019
TCS	23 (6.4%)	22 (17.3%)	<0.001
TCS Duration (median)	4.0 (IQR = 2.0)	6.0 (IQR = 4.0)	0.022
IABP	171 (47.2%)	71 (55.9%)	0.093
IABP Duration (median)	1.0 (IQR = 1.0)	2.0 (IQR = 3.0)	0.012

LVEF is expressed as mean + standard deviation. Other continuous variables were expressed as median with interquartile range

### Operative characteristics and postoperative outcomes

Approximately a quarter of the patients underwent redo-sternotomy with no difference in redo-sternotomies between the 2 groups (*P* = 0.220, Table 4). There was no difference in the incidence of concomitant procedures, duration of CPB, or LVADs (*P* > 0.05). There was no difference in the distribution of valvular regurgitant pathology between the 2 groups (Table 3). There was also no difference in concomitant valvular operations in the 2 groups (Table 4, *P* > 0.4). Mitral valve intervention was uncommon in our practice as its utility has remained unclear. Aortic valve intervention was performed for moderate to severe AI. Tricuspid valve procedures to improve competence was used for moderate to severe TR. In the low and high WBC groups, the proportion of patients with moderate or greater TR was 42.3 and 44.9% respectively. Moderate TR in the low and high WBC group were 28.1 and 33.9% respectively. For the high and low WBC groups respectively, there were no differences in the proportion of intrapericardial (55 (43.3) vs 143 (39.5%)) or preperitoneally (72 (56.7%) vs 219 (60.5%)) placed LVADs (*P* = 0.452).

**Table 4:** Operative Procedures and Parameters

	WBC < 10.5 K/μL (*n* = 362)	WBC > 10.5 K/μL (*n* = 127)	*P* Value
Redo-Sternotomy	103 (28.5%)	29 (22.8%)	0.220
Cardiopulmonary Bypass (min)	83.96 + 31.61	87.65 + 35.348	0.273
Sternum Closed	213 (58.8%)	82 (64.6%)	0.211
Chest Open (median days)	1.0 (IQR = 1.0)	1.0 (IQR = 1.0)	0.808
*Concomitant Surgery*			
Valve procedure	161 (44.5%)	54 (42.5%)	0.702
AV procedure	27 (7.5%)	6 (4.7%)	0.291
TV Procedure	143 (39.5%)	49 (38.6%)	0.855
MV Procedure	2 (0.6%)	0 (0.0%)	0.401
Intrapericardial LVAD	143 (39.5%)	55 (43.3%)	0.452
Preperitoneal LVAD	219 (60.5%)	72 (56.7%)	0.452

Cardiopulmonary bypass time was expressed as mean + standard deviation. Chest open days is expressed as median with interquartile range

**Fig. 1:**
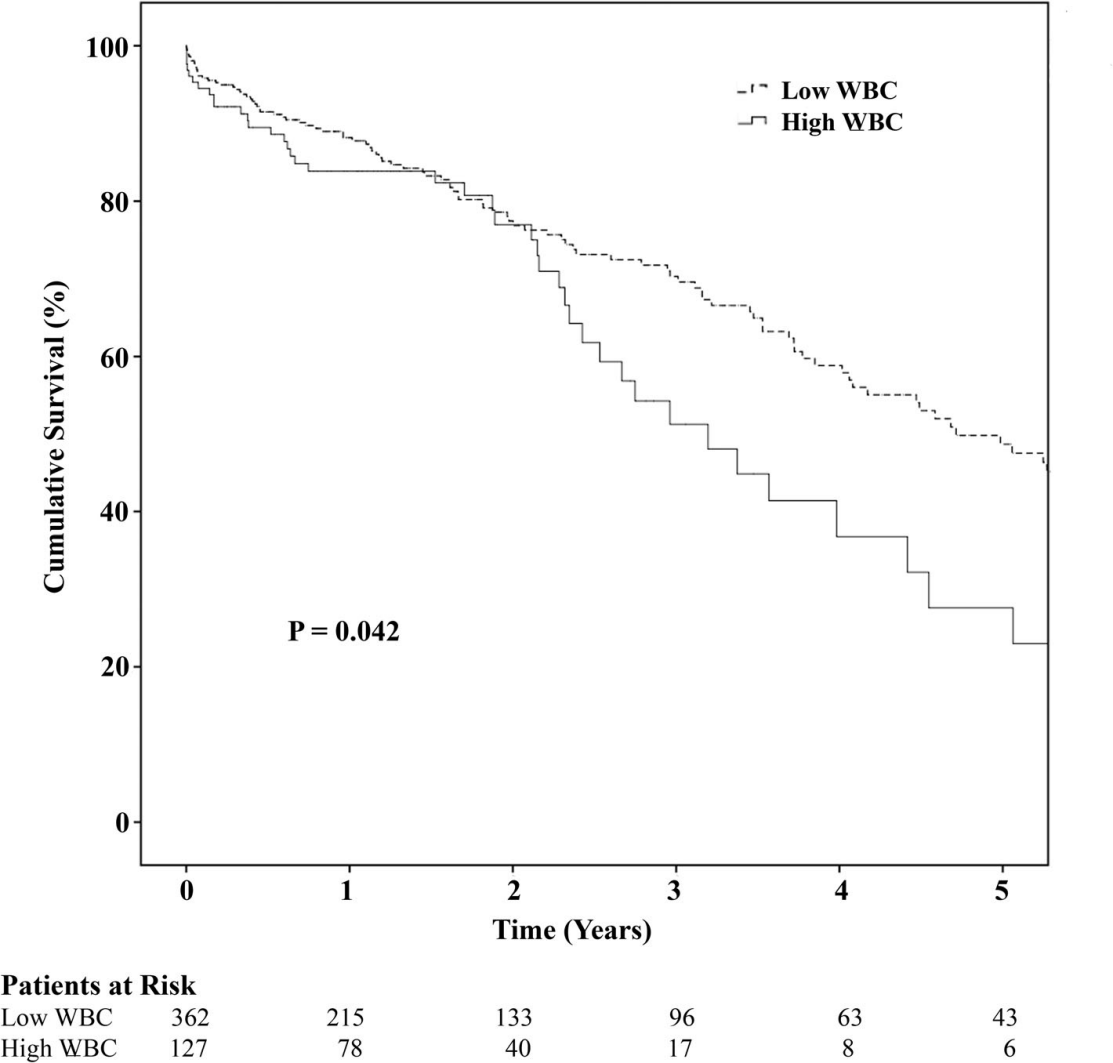
Kaplan-Meier Survival for patients with low and high WBC pre-LVAD implantation

The high WBC group (Table 5) had a longer length of intensive care unit (ICU, *P* = 0.046) and a trend toward longer total hospital stay (*P* = 0.064). There was more postoperative renal failure requiring dialysis in the high WBC group (*P* = 0.014), but no difference in subsequent permanent dialysis (*P* = 0.611). Postoperatively (Table 5), the higher WBC was more likely to experience right heart failure (13.4% vs 5.8%, *P* = 0.006), and need for a right ventricular assist device (RVAD) (11.0% vs 4.4%, *P* = 0.008). Patients experiencing postop RVF had a higher preop WBC (11.3 + 5.7 vs 8.7 + 3.1) and CRP (5.6 + 4.4 vs 3.3 + 4.7) levels. For those who needed an RVAD, there was no difference in the duration of use (*P* = 0.452). There was also no difference in the incidence (*P* = 0.950) and duration (*P* = 0.514) of nitric oxide use (Table 5). After excluding patients who underwent preoperative TCS, patients who had postoperative RVF had a higher preoperative WBC (10.3 + 4.4 vs 8.5 + 2.7, *P* = 0.002) and CRP (5.3 + 4.3 vs 2.9 + 4.2, *P* = 0.014) compared to those who did not. We performed a “Receiver Operating Characteristics” analysis and shows that WBC and CRP was able to predict RVF with a C-statistics of 0.661 (*P* = 0.001) and 0.727 (*P* < 0.001) respectively. There was no difference in other complications or operative mortality (*P* > 0.05). The high WBC group had poorer mid-term survival (Fig. 1, *P* = 0.042) with survival between the 2 groups diverging at approximately 2 years after cfLVAD implantation.

**Table 5:** Postoperative Outcomes

	WBC < 10.5 K/μL (*n* = 362)	WBC > 10.5 K/μL (*n* = 127)	*P* Value
Total ICU LOS (mean days)	10.95 + 17.22	15.73 + 34.92	0.046
Total LOS (mean days)	26.06 + 20.15	30.86 + 35.58	0.064
Total Days Readmit (mean days)	38.14 + 54.11	31.94 + 45.47	0.248
Device Infection	95 (26.2%)	26 (20.5%)	0.195
Device Exchange Infection	21 (5.8%)	7 (5.5%)	0.904
Late AV Intervention	5 (1.4%)	1 (0.8%)	0.601
Stroke	94 (26.0%)	32 (25.2%)	0.864
Hemolysis	87 (24.0%)	28 (22.0%)	0.650
Postop Dialysis/CVVH	12 (3.3%)	11 (8.7%)	0.014
Postop Perm Dialysis	6 (1.7%)	3 (2.4%)	0.611
LVAD Death	107 (29.6%)	41 (32.3%)	0.565
Transplant	132 (36.5%)	42 (33.1%)	0.492
Operative Mortality	16 (4.4%)	8 (6.3%)	0.399
RV Failure	21 (5.8%)	17 (13.4%)	0.006
RVAD	16 (4.4%)	14 (11.0%)	0.008
Duration of RVAD (median)	17.0 (IQR = 18.0)	20.50 (IQR = 74.0)	0.452
Nitric Oxide Use	356 (98.3%)	125 (98.4%)	0.950
Nitric Duration (median days)	2.0 (IQR = 2.0)	2.0 (IQR = 2.0)	0.514

Hospital stay times are expressed as mean + standard deviation. Duration of RVAD and nitric oxide use is expressed as median with interquartile range

## Discussion

Our studies demonstrate that in patients undergoing cfLVAD implantation, those who have a higher WBC preoperatively had a poorer postoperative and long term prognosis. The CRP, another commonly measured lab value, was also elevated consistent with a proinflammatory environment. While there was no difference in creatinine, there were laboratory evidence of liver dysfunction with elevations in total bilirubin and INR in the high WBC group. Higher WBC was also associated with a greater severity of heart failure with a greater proportion of patient classified as INTERMACS Patient Profile 1, higher heart rate, lower right ventricular stroke work index, as well as a greater incidence and duration of preoperative TCS. The high WBC group may be younger because more of these patients presented with acute cardiogenic shock and received temporary TCS with subsequent LVAD as bridge to transplant. Conversely, the older group were more likely to have chronic heart failure treated with destination therapy LVAD without the acute shock presentation requiring TCS. Furthermore, younger patient are more likely to mount a greater inflammatory response to cardiovascular stress in the LVAD setting as immunosenescence and dysfunction of the immune system progresses with aging [12].

The higher WBC may be attributed to the greater underlying acuity with likely more inotropic support as well as need for TCS preoperatively. Blood contact with artificial surfaces during pre-cfLVAD mechanical support induces an increase in chemokines and cytokines such as IL-8, MCP-1, MIP-1β, IP-10, GM-CSF [13]. This elevated preoperative inflammatory milieu in our study population is correlated with a greater incidence of RVF (5.8% vs 13.4%, *P* = 0.006) and need for RVAD (4.4% vs 11.0%) postoperatively. Cytokines such as TNF-α and its receptor has been shown to be upregulated in the failing myocardium [14]. Its role in causing myocardial dysfunction has been demonstrated where TNF-receptor-associated factor 2 (TRAF2) expression lead to heart failure, and this can be reversed after removal of inflammatory stimulus [15]. Furthermore, increased TNF-α levels are associated with greater nonsurgical bleeding following LVAD implantation [16].

Many previous studies have focused on postoperative inflammatory status on prognosis. Inflammatory cytokines were higher in nonsurvivors over 30 days post-LVAD implantation. In particular TNF-α, IL-1β, and IL-8 were elevated within hours to 7 days postoperatively [17]. Our finding that the high WBC group had a higher ICU and total hospital length of stay is consistent with prior findings where patients with higher preoperative IL-6, IL-8 and neopterin levels had a longer ICU stay and a greater decline in end organ function [18]. This impact on end organ dysfunction is reflected in our study where higher preoperative WBC was associated with a greater incidence of postoperative dialysis.

Interestingly, the high WBC group also had a greater proportion of patients that were bridged to transplantation consistent with a more severe heart failure with likely a higher incidence of biventricular dysfunction. Furthermore, our study showed that elevation in pre-cfLVAD WBC has long terms implications with a poorer mid-term survival that becomes evident 2 years after cfLVAD implantation. Late RVF may be a possible explanation for this finding.

The association of a preoperative proinflammatory state (elevated WBC and CRP) with the occurrence of postoperative RVF provides insights into the clinical relevance of inflammation in impacting myocardial contractility. Given the limited predictive value of current post-LVAD RVF prediction models, preoperative inflammatory markers may need to be incorporated into the algorithm for improved accuracy. It is also possible that suppression of excessive preoperative inflammation can decrease the incidence of RVF and need for RVAD.

This study has limitations due to its retrospective nature in a single institution with inherent limitations and biases. Elevation in WBC may reflect a higher INTERMACS status with greater inotrope use, use of TCS and the stress of a greater degree of heart failure. It remains unclear whether an independent elevation in WBC reflecting a greater inflammatory status is causative for poorer postoperative and long term outcomes. We cannot definitively exclude the likelihood of infection playing a role in elevated WBC since only a minority of patients have microbiological work up to exclude this possibility. Furthermore, it is possible that steroids used to treat inflammatory cardiomyopathies (e.g. giant cell myocarditis) preoperative may have contributed to elevated WBC. While there was a difference in age, high WBC group was younger but still had a poorer survival despite this. We acknowledge that there are multiple preoperative factors that may cause elevations in WBC and CRP, this introduces heterogeneity into our study groups.

## Conclusions

Our study demonstrates that a preoperative proinflammatory milieu characterized by elevations of WBC and CRP can be associated with postoperative RVF. Increased WBC was also associated with a longer length of hospital and ICU stay, a higher incidence of postoperative dialysis, as well as poorer long term patient survival. Preoperative proinflammatory stimuli likely include temporary mechanical support, catecholamines, concurrent infection, and low cardiac output syndrome with tissue ischemia. Leukocytosis is likely a marker of tissue stress and hypoperfusion. Furthermore, it is well recognized that inflammation can have a negative impact on myocardial contractility with implications for RVF after LVAD implantation.

## Abbreviations

BMI: Body Mass Index
cfLVAD: Continuous Flow Left Ventricular Assist Device
CPB: Cardiopulmonary Bypass
Cr: Creatinine
CRP: C-Reactive Protein
CRT-D: Cardiac Resynchronization Therapy-Defibrillator
IABP: Intra-Aortic Balloon Pump
ICU: Intensive Care Unit
INR: International Normalized Ratio
LDH: Lactate Dehydrogenase
LVAD: Left Ventricular Assist Device
MCS: Mechanical Circulatory Support
RVAD: Right Ventricular Assist Device
RVD: Right Ventricular Dysfunction
RVF: Right Ventricular Failure
SOFA: Sequential Organ Failure Assessment
TCS: Temporary Circulatory Support
WBC: White Blood Count
